# Microbiota dynamics preceding bariatric surgery as obesity treatment: a comprehensive review

**DOI:** 10.3389/fnut.2024.1393182

**Published:** 2024-04-03

**Authors:** Ana Karina Zambrano, Elius Paz-Cruz, Viviana A. Ruiz-Pozo, Santiago Cadena-Ullauri, Rafael Tamayo-Trujillo, Patricia Guevara-Ramírez, Raynier Zambrano-Villacres, Daniel Simancas-Racines

**Affiliations:** ^1^Facultad de Ciencias de la Salud Eugenio Espejo, Centro de Investigación Genética y Genómica, Universidad UTE, Quito, Ecuador; ^2^Universidad Espíritu Santo, Samborondón, Ecuador; ^3^Centro de Investigación de Salud Pública y Epidemiología Clínica (CISPEC), Universidad UTE, Quito, Ecuador

**Keywords:** bariatric surgery, gut microbiota, obesity, microbiota dynamics, obesity treatment

## Abstract

The review present data on the intricate relationship between bariatric surgery, gut microbiota, and metabolic health in obesity treatment. Bariatric surgery, is recognized as an effective intervention for managing morbid obesity, including various techniques with distinct mechanisms of action, efficacy, and safety profiles including Roux-en-Y Gastric Bypass (RYGB), Sleeve Gastrectomy (SG), Laparoscopic Adjustable Gastric Banding (LAGB), and Biliopancreatic Diversion (BPD). RYGB and SG are the most prevalent procedures globally, inducing gut microbiota changes that influence microbial diversity and abundance. Post-surgery, alterations in bacterial communities occur, such as the increased of *Escherichia coli* inversely correlated with fat mass and leptin levels. During digestion, microbiota produce physiologically active compounds like bile acids (Bas) and short-chain fatty acids (SCFAs). SCFAs, derived by microbial fermentation, influence appetite, energy metabolism, and obesity-related pathways. Bas, altered by surgery, modulate glucose metabolism and insulin sensitivity. Furthermore, SG and RYGB enhance incretin secretion, particularly glucagon-like peptide 1 (GLP-1). Therefore, understanding microbiota changes after bariatric surgery could be crucial for predicting metabolic outcomes and developing targeted interventions for obesity management.

## Introduction

The treatment of obesity presents numerous challenges due to its complex and multifactorial nature. In recent years, this condition has emerged as a global epidemic. In response to this growing health crisis, bariatric surgery has emerged as an effective intervention for managing morbid obesity and its associated comorbidities (1–3). Bariatric surgery modifies the gastrointestinal system to alter nutrient absorption (malabsorptive mechanisms) and/or restrict food intake (restrictive mechanisms) as an approach for weight management. Candidates for this surgical intervention typically have a body mass index (BMI) of 40 kg/m^2^ or higher, or a BMI of 35 kg/m^2^ or higher with significant comorbidities (3).

Various techniques are employed in bariatric surgery, each with its unique mechanism of action and associated considerations. Among the most commonly utilized techniques are biliopancreatic diversion (BPD), adjustable gastric banding (LAGB), sleeve gastrectomy (SG), and Roux-en-Y gastric bypass (RYGB). The selection of the appropriate bariatric procedure depends on several factors including the severity of obesity, presence of comorbidities, and patient preferences (4).

### Roux-en-Y gastric bypass (RYGB)

RYGB stands as one of the oldest and most popular bariatric procedures, ranking as the second most common worldwide (5). During this procedure, significant portions of the stomach and proximal small intestine are bypassed, resulting in the creation of a small gastric pouch directly connected to the small intestine. RYGB achieves weight loss by combining malabsorptive and restrictive mechanisms (6).

Studies consistently demonstrate that RYGB leads to substantial weight loss and improvement in associated comorbidities such as type 2 diabetes and hypertension (7, 8). However, RYGB poses inherent risks including intestinal blockage, internal hernia, and long-term metabolic issues stemming from nutrient malabsorption. Despite these risks, RYGB remains popular due to its proven effectiveness in promoting weight loss and metabolic health (5, 8, 9).

### Sleeve gastrectomy (SG)

SG has gained prominence in recent years due to its less invasive nature compared to RYGB, while also preserving normal stomach function. Currently, it stands as the most common bariatric surgery (10, 11). This procedure involves the removal of a large portion of the stomach, leaving behind a narrow gastric tube that restricts food intake (10).

Both SG and RYGB yield similar outcomes in terms of weight loss and improvement in metabolic comorbidities. Additionally, SG, not requiring intestinal anastomosis, may be associated with a lower incidence of long-term complications such as intestinal blockage and internal hernia. Nevertheless, SG is not without risks, with potential postoperative issues including staple line leaking and stomach stricture (12).

### Laparoscopic adjustable gastric banding (LAGB)

LAGB is a restrictive procedure wherein a silicone band is placed around the stomach to create a small upper gastric pouch. Unlike RYGB and SG, LAGB offers reversibility and adjustability, making it an attractive option for certain patients (13).

Although LAGB was previously popular due to its reversible and minimally invasive nature, its utilization has declined in recent years due to lower rates of sustained weight reduction and increased risk of long-term complications such as band slippage and stomach erosion (14, 15).

### Biliopancreatic diversion (BPD)

BPD combines gastric bypass with distal gastrectomy, resulting in a reduced intestinal absorption area and nutritional malabsorption (16). While BPD is highly effective for weight loss and improving metabolic comorbidities, it also raises the risk of long-term complications such as protein and vitamin deficiencies (17, 18).

The primary objective of the present mini review is to describe the most prevalent bariatric surgery techniques used in the treatment of obesity, including RYGB, SG, LAGB, and BPD. The manuscript explores how these surgical procedures alter the complex environmental community of microorganisms, part of our microbiota, and the effect that these changes have on human health and obesity. Furthermore, the article discusses the clinical aspects of bariatric surgery, including weight loss outcomes, post-operative complications, and contributions to metabolic health enhancement. It emphasizes the importance of the gut microbiota composition in weight management, influencing lipid metabolism, hormone signaling, and glucose homeostasis.

## Bariatric surgery as treatment for obesity

According to the World Health Organization, more than 650 million adults were obese in 2016, with a perspective of an increment (19). Besides, obesity is associated with elevated risk of various comorbidities, including type 2 diabetes, heart conditions, hypertension, among others (20). Consequently, bariatric surgery has emerged as an effective and long-lasting option for achieving significant health improvements.

Bariatric surgery is considered a potential treatment intervention for obesity, commonly for subjects with a BMI of 40 kg/m^2^ or higher, or a BMI of 35 kg/m^2^ or higher with significant comorbidities. The primary goal of bariatric surgery is to reduce the size of the stomach or alter the digestive tract to induce the decrease the volume of food taken and its absorption, with an impact from hormonal to molecular changes (21, 22). Consequently, bariatric surgery can result in a 50–70% in short term weight loss or 20–30% loss of the patient's initial weight (23).

The mechanisms of action, effectiveness, and safety profiles vary among the different types of bariatric surgery. The RYGB and SG are the most common procedures worldwide, accounting for 72,645 individuals (38.2%) and 87,467 individuals (46%) of all primary operations since 2014, respectively. On the other hand, One-anastomosis gastric bypass (OAGB) and gastric bypass (GB) surgeries are less frequently performed, representing 14,516 individuals (7.6%) and 9,534 individuals (5%) of all primary operations since 2014, respectively (24, 25). After one-year post-surgery, the mean weight loss was 28.9% with an improving in metabolic health. Remarkably, 66.1% of patients with type 2 diabetes did not need more medication. Consequently, the degree of diabetes remission correlated closely with weight loss achieved (25).

Despite the significant benefits of bariatric surgery, it is crucial to acknowledge potential complications associated with these procedures. Data from clinical studies and registry analyses into the rates of postoperative complications are limited. However, a systematic review reported that the most common complications within 30 days after bariatric procedures, includes anastomotic leak, myocardial infarction, pulmonary embolism (26). Another study reported that the most common complication after surgery is peritonitis with an incidence of 1–6% after GB and 3–7% after SG (27). The most frequent late postoperative complications are dumping syndrome and cholecystitis, each occurring in up to 30% of cases (27). Additionally, the perioperative mortality rate is <1% (27). Despite advancements in surgical techniques and perioperative care leading to improvements in safety outcomes, it is essential for healthcare providers and patients to be aware of the potential risks associated with bariatric surgery.

Minimally invasive approaches, such as laparoscopic and robotic-assisted procedures, have become increasingly common in bariatric surgery. These techniques result in shorter hospital stays, faster recovery times, and improved patient outcomes. Additionally, multidisciplinary care involving nutritionists, psychologists, and other healthcare professionals plays a crucial role in mitigating complications and supporting patients throughout their bariatric surgery (28). Furthermore, studies suggest that individuals with obesity who undergo bariatric surgery should engage in moderate physical activity and make dietary changes to sustain their weight loss (29, 30).

Consequently, bariatric surgery is a noteworthy treatment for obesity, offering significant weight loss outcomes and improvements in metabolic health. While RYGB and SG continue to be the predominant surgical modalities, ongoing research and advancements in surgical techniques aim to further enhance the safety and efficacy of these procedures.

## The relationship between obesity and microbiota

Recent research has elucidated the intricate relationship between obesity and the gut microbiota, particularly focusing on the modulation of host metabolism by microbiota-derived metabolites. Short-chain fatty acids (SCFAs), such as acetate, propionate and butyrate, are produced through the fermentation of dietary fiber by gut bacteria. SCFAs act as a signaling molecules influencing metabolic processes crucial for energy homeostasis and lipid metabolism (31, 32). Furthermore, the gut microbiota impacts host metabolism by regulating the expression of genes involved in adipogenesis, lipid metabolism, and insulin sensitivity. Dysbiosis, observed in obese individual, contributes in disturbances involving host-microbiota interactions.

Obesity is associated with chronic low-level inflammation in several tissues, which has been correlated with metabolic diseases like type 2 diabetes, insulin resistance, and cardiovascular diseases. One of the effects of this chronic inflammation is gut barrier impairment. It has been proposed that hyperglycemia and a low gut bacteria diversity could lead to gut barrier permeabilization, allowing the entry of antigenic compounds like lipopolysaccharides to blood circulation. These antigenic compounds can induce endotoxemia, insulin resistance and chronic immune system activation (33). Therefore, modulating gut microbiota after bariatric surgery could potentially improve the intestinal barrier and restore metabolic homeostasis (33).

Emerging evidence suggests the role of gut microbiota in obesity. Studies comparing the microbial communities in obese individuals with non-obese, have consistently revealed differences in the abundance Firmicutes and Bacteroidetes (34–36). Another study demonstrated an increase in *Bacteroidetes thetaiotaomicron*, a glutamate fermenting commensal, in obese individuals who follows a weight-loss intervention (like sleeve gastrectomy) (37). Consequently, *B. thetaiotaomicron* reduces plasma glutamate concentration and may protect against body weight gain induced by diet and adiposity (37). Moreover, research has shown that *Bacteroidetes uniformis* relieves high-fat-diet induced obesity, complementing the effect of *B. thetaiotaomicron* (38). Specifically, *B. uniformis* increases TNF-α production by dendritic cells (DCs) in response to purified lipopolysaccharide stimulation (reduced by high-fat-diet) (38).

Consequently, the increased abundance and diversity of SCFA-producing bacteria, lead to heightened production of host glucagon-like peptide 1 (GLP-1), which contributes to glucose-dependent stimulation of insulin secretion, inhibition of food intake, increase of natriuresis and diuresis, among other metabolic effects (39). Dysregulation of these pathways may contribute to overeating and weight gain, highlighting the molecular basis of the gut-brain axis in obesity (40). Furthermore, the gut microbiota produces metabolites that play critical roles in lipid metabolism, energy expenditure, and inflammation, thus shaping the metabolic phenotype of the host (31, 41, 42). Strategies aimed at modulating gut microbiota composition and activity, such as probiotics, prebiotics, and fecal microbiota transplantation, have shown potential in improving metabolic dysfunction associated with obesity.

Furthermore, the consumption of the probiotic *Lactobacillus gasseri BNR17* has been approved by the Korean FDA as an ingredient to reduce visceral adipose tissue in adults with obesity (43). Moreover, some studies are based on *Akkermansia muciniphila, Faecalibacterium prausnitzii* and *Clostridia strains* considered as possible probiotics, most of them present in the human intestinal microbiota. These strains produce butyrate and other short-chain fatty acids (SCFAs), compounds that are decreased in people with obesity (44).

## Microbiota changes preceding bariatric surgery

Recent studies have highlighted the significant role of the gut microbiota before and after bariatric surgery, associating the complex interplay between gut microbiota, surgical interventions, and metabolic health outcomes (Table 1). A ten-year review study described the alterations in gut microbiota composition in obese individuals before and after bariatric surgery, showing that changes in the composition and function of gut microbiota affect metabolic functions in obese patients, leading to significant physiological regulation (50).

**Table 1:** Differential changes in gut microbial composition post-bariatric surgery.

**Types of bariatric surgery**	**Bacteria**	**Abundance**	**Outcome**	**References**
RYGB	*Bacteroides*	Increase	These changes occurred after surgery and were inversely correlated with fat mass and leptin levels.	(45)
	*Prevotella*	Increase		
	*Escherichia coli*	Increase		
	*Lactobacillus*	Decrease		
	*Leuconostoc*	Decrease		
	*Pediococcus*	Decrease		
	*Enterobacter cancerogenus*	Increase	These changes improved host lipids and glucose levels.	(46)
	Firmicutes	Decrease		
	Bacteroidetes	Decrease		
	Proteobacteria	Increase	The fecal profiles reflected an increased activity of oligosaccharide fermentation in the gut and the generation of amines, which may contribute to body weight loss.	(47, 48)
	*Bacteroides thetaiotaomicron*	Decrease		
SG	Bacteroidetes/ Firmicutes ratio	Decrease	The capacity for butyrate fermentation decreased. This could be attributed to changes in the abundance of Firmicutes	(45)
	*Akkermansia muciniphila*	Increase	The increase in this species after surgery is related to better glucose homeostasis and lipid metabolism.	(49)
	Bacteroidetes	Increase	These changes in microbial abundance after surgery play a role in reducing low-grade inflammation.	(49)
	Firmicutes	Decrease		

RYGB, Roux-en-Y gastric bypass; SG, sleeve gastrectomy.

The type of bariatric surgery performed influences the changes in gut microbiota. Several studies have investigated changes following RYGB, noting variation in microbial diversity across intestinal segments after surgery (51). Furthermore, other studies have reported the changes in the microbial communities. For example, one study found that the *Bacteroides/Prevotella* was lower in obese subjects and increased post-surgery. Additionally, lactic and acid bacteria (*Lactobacillus, Leuconostoc, Bifidobacterium, and Pediococcus group*) were reduced, while *Escherichia coli* increased after surgery and inversely correlated with fat mass and leptin levels independent of dietary changes (45). Another study reported that after RYGB, *Enterobacter cancerogenu*s (*Proteobacterium*) increased, while Firmicutes and Bacteroidetes decreased, improving in host lipids and glucose levels (46). Similarly, other studies also reported a decrease in Firmicutes and Bacteroidetes (specifically *Bacteroides thetaiotaomicron)* after surgery while Proteobacteria species increased (47, 48).

Studies on SG, also found significant shifts in the gut microbiota. A next -generation sequencing analyses revealed a decrease in energy-reabsorbing potential after SG, indicated by the Bacteroidetes/Firmicutes ratio. Additionally, the capacity for butyrate fermentation decreased, attributed to Firmicutes changes (52).

The impact of microbial shifts extends beyond weight loss. For instance, the increase of *Akkermansia muciniphila* post-surgery is linked to improved glucose homeostasis and lipid metabolism critical in the remission of type 2 diabetes. Besides, the decrease in Firmicutes and increase in Bacteroidetes after surgery play a role in reducing low-grade inflammation associated with obesity and metabolic syndrome (49).

**Figure 1:**
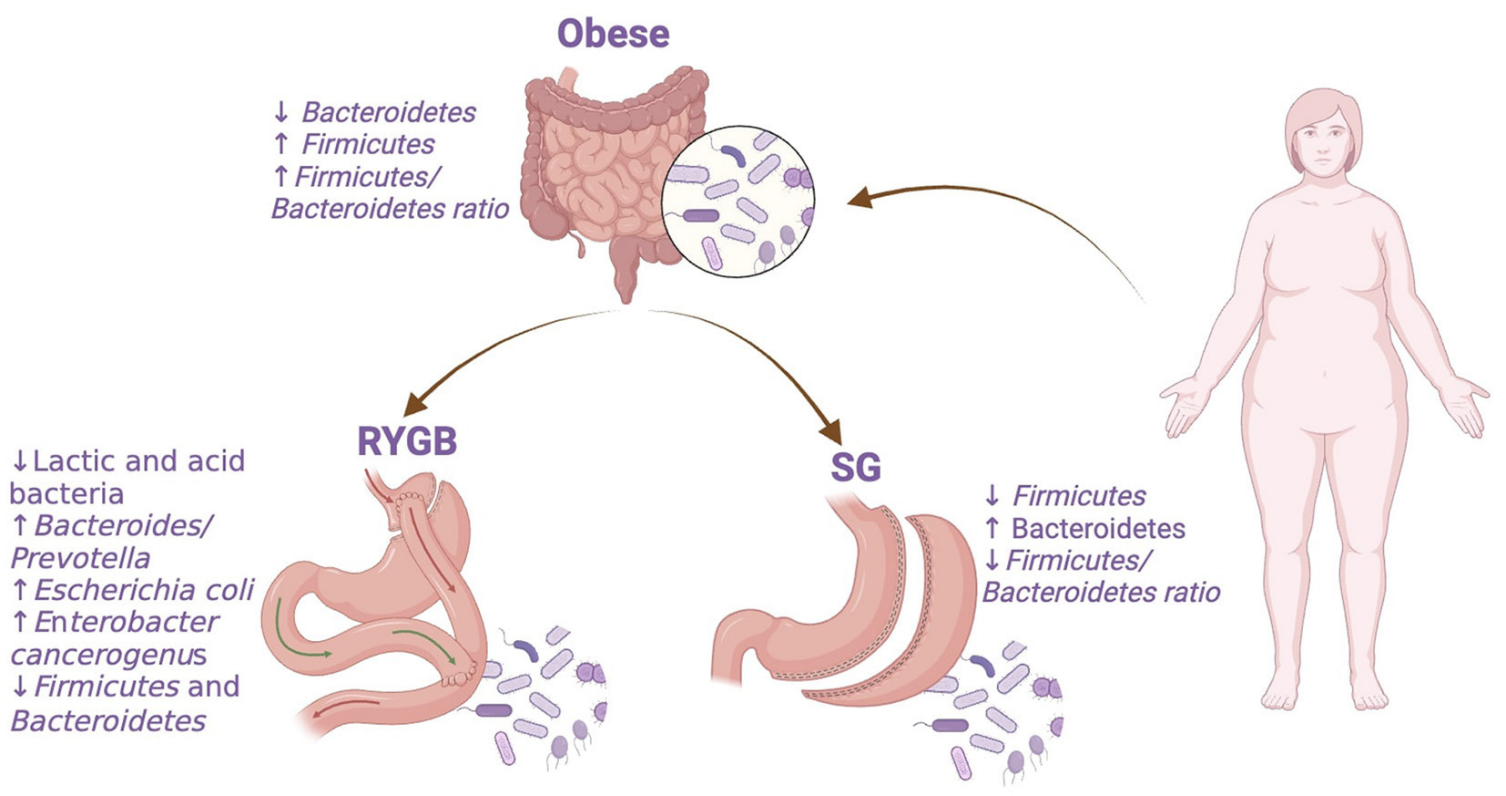
Bacterial abundance changes in obese individuals before and after bariatric surgery. RYGB, Roux-en-Y gastric bypass; SG, sleeve gastrectomy. The figure was created with BioRender.com.

Understanding these bacterial changes (Figure 1) is essential for predicting the bariatric surgery outcomes. However, future research may focus on preoperative and postoperative modulation of the gut microbiota to enhance bariatric procedures.

## Discussion

The human intestine harbors over 100 trillion microbial cells, which significantly influences metabolic regulation through symbiotic interactions with the host (53). During digestion, the gut microbiota generates various physiologically active compounds, including BAs, SCFAs, and long-chain fatty acids (LCFAs) (31). The activity and composition of the gut microbiota can be altered by various factors such as nutritional intake, gastric emptying, and gastric acid production, thereby being influenced by different bariatric surgical methods (54, 55).

The chronic inflammatory process observed in various tissues of individuals with obesity has been linked to gut mucosa impairment. This impairment is associated with reduced synthesis of the mucus protein layer, allowing the release of pro-inflammatory cytokines (IL-1b, IL-6, IL-12, IL-18). Bariatric surgery has been related to an improved intestinal barrier synthesis due to the expression of ZO-1, occludin, and claudin-1 tight junction proteins. Moreover, the restoration of bacteria involved in SCFA synthesis, which has been observed after bariatric surgery (33), has also been linked to intestinal barrier restoration (56). Therefore, the restoration of intestinal mucosa layer could potentially improve the inflammatory process and restore the metabolic homeostasis.

Among the bariatric procedures, SG and RYGB stand out as the most widely practiced, both contributing to an increase in the secretion of incretins by augmenting the number of secreting cells, particularly GLP-1 (57).

Short-chain fatty acids, including butyrate, propionate, and acetate, are key metabolites derived from the metabolism of complex carbohydrates by gut microbiota (58). While studies indicate higher fecal concentrations of SCFAs in obese individuals, their role in energy metabolism and obesity remains controversial due to their dual effects on hunger reduction, lipogenesis inhibition, and induction of browning in white adipose cells (40, 59, 60). SCFAs exert their appetite-suppressing effects by interacting with isolated neurons in the nodal ganglia, triggering intracellular Ca^2+^ signaling, and elevating serum levels of leptin, GLP-1, and peptide YY (PYY) (61, 62).

Following bariatric surgery, fecal SCFA levels decrease, primarily attributed to low-carbohydrate diets, possibly indicating inefficient utilization of dietary SCFAs for energy during weight loss (63). RYGB surgery reduces stomach acid secretion, leading to higher levels of partially digested proteins in the intestine and resulting in putrescine generation. Additionally, an increase in *Klebsiella* bacteria post-RYGB further contributes to putrescine production. The metabolism of putrescine yields gamma-aminobutyric acid (GABA), exacerbating insulin resistance and elevating GLP-1 levels (64).

After RYGB, there is an increase in the abundance of *Streptococcus, Veillonella*, and *Akkermansia* species. *Streptococcus* and *Veillonella* metabolize lactate, impacting butyrate metabolism and epithelial barrier integrity, which may potentially ameliorate metabolic disorders, and reduce systemic inflammation. *Akkermansia muciniphila*, has been associated with protection against diabetes and obesity in animal studies, may improve insulin sensitivity and reduce inflammation, further enhancing intestinal epithelial integrity in humans (65, 66).

Bariatric surgery induces alterations in BA metabolism, enhancing energy homeostasis. BAs play an important role in gut microbiota composition and post-surgery weight loss by modulating glucose metabolism, increasing insulin sensitivity, and reducing gluconeogenesis through elevated GLP-1 secretion and activation of G protein-coupled receptor (TGR5) and nuclear receptor (FXRα) pathways (67–70).

Ilhan et al. (71) reported decreased fecal BA concentration in obese patients post-RYGB. This decrease was associated with microbiota composition changes (71). RYGB-induced architectural modifications enhance the BA influx into the lower intestine. This facilitates reabsorption of conjugated BAs in the terminal ileum and conversion of primary to secondary BAs by gut microbes in the colon. These metabolic improvements, including gut microbiota repopulation and altered primary/secondary BAs ratio, had a positive impact on metabolic syndrome (45, 72).

In contrast, Evers et al. (73) observed decreased levels of lithocholic acid (LCA) in the colon and increased levels in the portal vein post-SG. LCA promotes CA7S production in the livers of mice and humans, impacting host metabolism. The researchers also determined that LCA activates the vitamin D receptor and induces cholic acid sulfonation both *in vitro* in human hepatocytes and *in vivo* in mice. The CA7S synthesized by LCA in human hepatocytes can trigger GLP-1 secretion in enteroendocrine cells, establishing a link between BA level alterations post-SG and the favorable effects on energy and glucose homeostasis (73).

## Conclusion

In conclusion, the comprehensive review of microbiota dynamics preceding bariatric surgery emphasizes the role of gut microbiota in the management of obesity and associated metabolic disorders. The observed alterations in gut microbial composition following bariatric procedures, such as Roux-en-Y gastric bypass and sleeve gastrectomy, highlight the potential for microbiota modulation as a therapeutic strategy to enhance surgical outcomes. These alterations, including changes in microbial diversity and abundance, have been linked to improvements in glucose homeostasis, lipid metabolism, and inflammation, crucial factors in achieving remission of type 2 diabetes and metabolic syndrome. Understanding the complex interplay between gut microbiota, surgical interventions, and metabolic health outcomes is essential for optimizing patient care and developing targeted interventions to enhance the efficacy of bariatric surgery. Further research into preoperative and postoperative microbiota modulation holds promise for improving the safety and long-term success of bariatric procedures, ultimately offering hope for individuals grappling with obesity and its related complications.

## Author contributions

AZ: Conceptualization, Investigation, Supervision, Writing – original draft, Writing – review & editing. EP-C: Conceptualization, Investigation, Writing – original draft, Writing – review & editing. VR-P: Investigation, Writing – review & editing. SC-U: Investigation, Writing – review & editing. RT-T: Investigation, Writing – review & editing. PG-R: Investigation, Writing – review & editing. RZ-V: Investigation, Writing – review & editing. DS-R: Investigation, Writing – review & editing.
